# The Impact of Different Degrees of Leadership on Collective Navigation in Follower-Leader Systems

**DOI:** 10.1007/s11538-025-01435-z

**Published:** 2025-04-07

**Authors:** Sara Bernardi, Kevin J. Painter

**Affiliations:** 1 Department of Mathematical Sciences “G. L. Lagrange”, Politecnico di Torino, Corso Duca degli Abruzzi 24, 10129 Turin, Italy; 2 Institute of Atmospheric Sciences and Climate, National Research Council of Italy, Corso Fiume 4, 10133 Turin, Italy; 3 Interuniversity Department of Regional and Urban Studies and Planning, Politecnico di Torino, Viale Pier Andrea Mattioli 39, 10125 Turin, Italy

**Keywords:** Leadership, Collective migration, Nonlocal PDEs, Group heterogeneity

## Abstract

In both animal and cell populations, the presence of leaders often underlies the success of collective migration processes, which we characterise by a group maintaining a cohesive configuration that consistently moves toward a target. We extend a recent non-local hyperbolic model for follower-leader systems to investigate different degrees of leadership. Specifically, we consider three levels of leadership: *indifferent* leaders, who do not alter their movement according to followers; *observant* leaders, who attempt to remain connected with the followers, but do not allow followers to affect their desired alignment; and *persuadable* leaders, who integrate their attempt to reach some target with the alignment of all neighbours, both followers and leaders. A combination of analysis and numerical simulations is used to investigate under which conditions each degree of leadership allows successful collective movement to a destination. We find that the indifferent leaders’ strategy can result in a cohesive and target-directed migration only for short times. Observant and persuadable leaders instead provide robust guidance, showing that the optimal leader behavior depends on the connection between the migrating individuals: if alignment is low, greater follower influence on leaders is beneficial for successful guidance; otherwise, it can be detrimental and may generate various unsuccessful swarming dynamics.

## Introduction

The expansion of Austronesian and Micronesian populations across the south Pacific demanded, quite naturally, considerable prowess for maritime navigation. Many navigating cues would have been used, including celestial bodies, characteristic wave and wind patterns, and probably others since forgotten. Following the paths of certain seabirds, such as terns, to their home islands provided an important source of information to mariners, pointing them in the direction of landmasses (Lewis 1994).

Follower-leader systems have attracted increasing levels of attention as a paradigm for certain collective movement processes, within both ecological (Berdahl et al. 2018; Brent et al. 2015; Strandburg-Peshkin et al. 2018) and cellular (Vishwakarma et al. 2018; Cheung et al. 2013; Vilchez Mercedes et al. 2021) populations. Simplistically, in a follower-leader driven process the leaders can be viewed as those members that establish and/or indicate a route that the followers can adopt. For example, leaders may be those with *a priori* information regarding the location of a destination, or through subtly different movement characteristics take up a more prominent position within the migrating group. However, while this partitioning into followers and leaders is conceptually straightforward, it remains a broad and indeterminate separation. In the opening example above, the leaders (seabirds) are highly distinct from the followers (mariners) and, most likely, indifferent to whether the followers remain within visual contact to successfully complete their navigation. In other instances, leaders may be highly vested in the migration success of the followers. African elephants matriarchs, for example, appear to provide leadership during group travel, offering guidance to water sources (Payne 2003). High-resolution GPS data reveal that dominant female meerkats exert greater influence than other individuals on both group direction and speed during foraging (Averly et al. 2022). The family structure inherent to such groups would exert a strong pressure to maintain group compactness, the leaders providing route guidance but also remaining attentive that followers stay on route. In other instances the distinction between followers and leaders may be even more subtle, for example within a population of relatively homogeneous individuals that differ in a phenotypic trait—for example, speed, “courage”, or ability to detect navigating cues—which leads them to adopt a more prominent/position role in the overall guidance (Sasaki et al. 2018). For example, a subtle leadership has been experimentally shown in shoals of sticklebacks, where the presence of partially informed individuals help them to find and access hidden food (Webster et al. 2017).

Numerous theoretical models have been developed to describe the collective movement of a population, ranging from agent-based models that track each individual’s movement path (e.g. Aoki 1982; Reynolds 1987; Vicsek et al. 1995; Couzin et al. 2002; Berdahl et al. 2018) to integro-partial differential equation models for evolving population density distributions (Eftimie 2018; Painter et al. 2024b). Unifying these varied approaches is the assumption that the movement dynamics are partly driven by various interactions between neighbouring individuals (Carrillo et al. 2010), typically some set of: individual to individual repulsion at short distances, e.g. to prevent collisions; individual to individual attraction at larger distances, e.g. to prevent group dispersal; and, individual to individual alignment, so that the group converges on a common orientation. Layered on top of this, external guidance cues may act to bias the overall movements towards some particular target (Codling and Bode 2016).

Recently, substantial research into collective behaviour has focused on the significance of population heterogeneity. Heterogeneity may occur at a heterospecific level—grazing groups formed from zebras, gazelles, wildebeest (Saltz et al. 2023)—or within the same species. At a cellular level, migrating cell populations may be composed by cells of multiple types, or cells of the same type but structured into distinct phenotypes with distinct motility and proliferative behaviours (Giese et al. 1996). In the context of heterogeneous groups composed from a mixture followers and leaders, it is logical to assume that the various orientation influencing factors described above will vary between the two populations. For example, in the context of mariners following seabirds, while it may be natural to assume that the mariners attract and/or align according to the observed positions and trajectories of birds, the birds are unlikely to respond similarly. In other follower-leader systems, where leaders “pay attention”, both followers and leaders may have some combination of attraction and alignment interactions according to all members of the population. It is also likely that leaders will differentiate themselves in other manners, such as through their capacity to detect and orient according to external guidance cues, adopting different speeds, or through controlling their level of conspicuousness, e.g. visibility to the follower. From a modeling perspective, the description of a broader range of follower-leader heterogeneity has been proposed using agent-based models. The balance between individual information and social influence among leaders in collective migration is studied in Guttal and Couzin (2010), where the authors also explore migratory strategies in response to environmental threats. In Couzin et al. (2011), both modeling theory and experiments are used to highlight the role of followers in achieving democratic consensus, particularly in the presence of internal group conflicts and informational constraints.

In this paper we consider an abstract framework that allows us to explore the extent to which different “degrees” of leadership can impact on collective migration processes. In particular, we build on a non-local hyperbolic framework of follower-leader dynamics formulated in Bernardi et al. (2021), which is in itself an extension of the model of Eftimie et al. (2007) to describe self-organisation and collective motility of a homogeneous population. Notably, this framework incorporates the various types of group-interaction described above, but through the continuous formulation remains somewhat amenable to formal analysis. In Bernardi et al. (2021) our focus was on the different mechanisms through which a leader population could impart directional information to a population of followers, but the leaders themselves were given a fixed set of interactions via which they interacted with the followers. Here we will relax that assumption and consider different types of leader.

The remainder of this paper is organized as follows. In the next section, we will describe the various types of leaders we will consider, and lay out the mathematical framework. In Sect. 3, we introduce and characterize the dynamics of the *indifferent* leader model. Sections 4 and 5 cover the formulation and resulting dynamics of the *observant* and *persuadable* leader models, respectively. We conclude with a discussion and suggestions for future investigation.

**Fig. 1:**
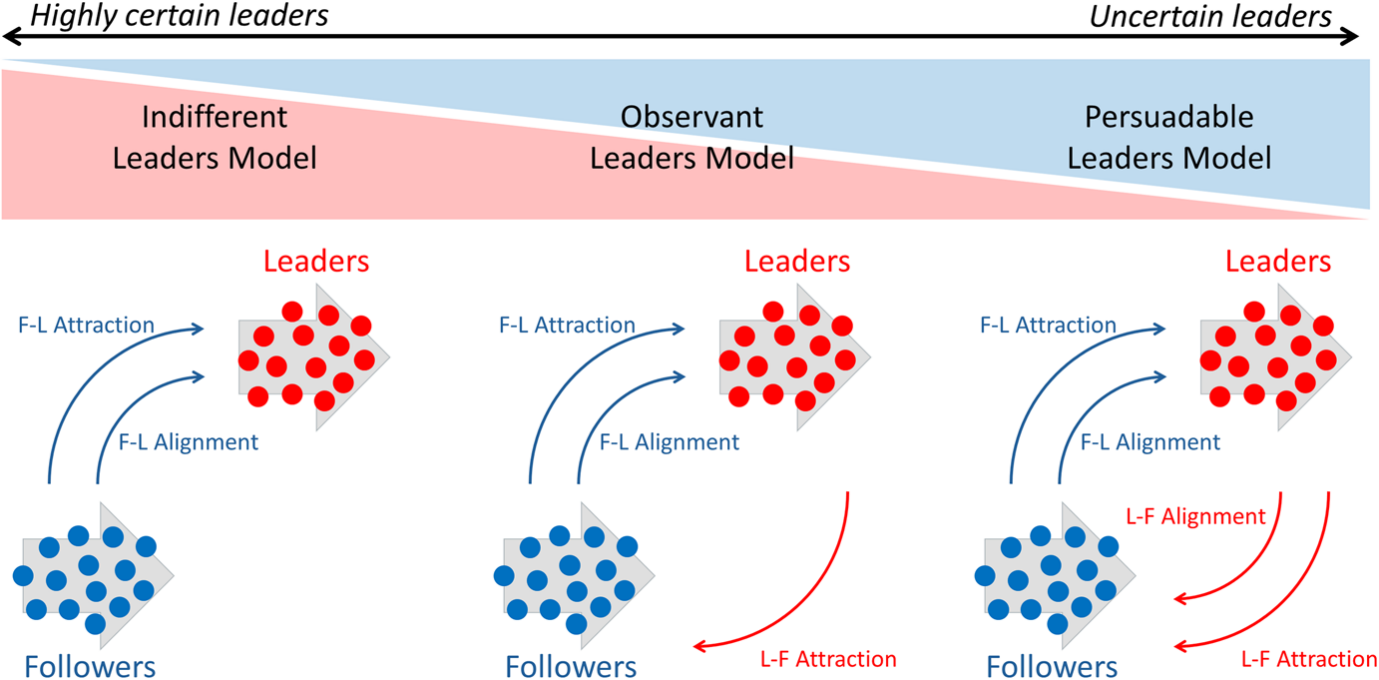
Modeling different degrees of leadership, ranging from highly certain leaders to uncertain leaders. *Indifferent* leaders have no movement response to followers; *observant* leaders respond to follower position through attraction; *persuadable* leaders respond to both follower position and alignment (Color figure online)

## Degrees of Leader and Model Structure

### Degrees of Leadership

As described in mathematical detail below, our intention is to formulate and analyse a model for follower-leader type collective guidance, where the movement dynamics of the two populations are determined by interactions between neighbouring individuals. Here we will restrict to a combination of

- *Attraction interactions*, in which individuals have a tendency to orient in the direction of higher population numbers. Attraction allows a swarm to form or maintain an aggregated state.
- *Alignment interactions*, in which individuals have a tendency to align according to the dominating alignment. Alignment can allow polarisation, or consensus for a specific direction.

As noted earlier, repelling interactions can also be considered within collective movement models, although we exclude these here for model compactness. We assume that leaders are aware of the target direction (e.g. from *a priori* knowledge or detecting a cue) but followers are *naïve*, and rely on the informed leaders. In previous work (Bernardi et al. 2021) we assumed that leaders were somehow invested in the success of the followers, focussing on different mechanisms for imparting route information. As such, the precise manner by which followers influenced leader behaviour was kept fixed. Here we relax this, in particular by considering *three leadership degrees*, see Fig. 1:

- *Indifferent* leaders. Followers have no influence on leader attractive or alignment responses. Thus, leaders are “indifferent” as to whether followers successfully follow.
- *Observant* leaders. Followers influence leader attraction responses. We interpret this as a leader population that will alter its movement in order to remain connected with followers, but do not allow followers to influence their own intended alignment.
- *Persuadable* leaders. Followers influence both leader attraction and alignment. This could reflect more “subtle” leaders that may have some information on the target, but can be swayed by the alignment choices of followers.

### Model Framework

To implement these models we utilise a nonlocal hyperbolic PDE approach, first developed in Eftimie et al. (2007) (see also Eftimie 2018). Here we briefly summarise the model structure for the case of a single (homogeneous) population; in subsequent sections we outline its adaption to describe the various follower-leader systems.

Consider a motile population, subdivided into its proportions moving right ($+$) or left (−), each with fixed speed $\gamma$. We set $p^+(x,t)$ and $p^-(x,t)$ to denote the population densities of the $+$ and − populations, respectively, at position $x \in \Omega \subset {\mathbb {R}}$ and time $t \in [0, \infty )$. We let $p(x,t)=p^+(x,t)+p^-(x,t)$ denote the total population density. The governing equations are as follows:

1 $$\begin{aligned} \frac{\partial p^+}{\partial t} + \gamma \frac{\partial p^+}{\partial x}= &  -\lambda ^{p^+} p^+ +\lambda ^{p^-} p^-, \nonumber \\ \frac{\partial p^-}{\partial t} - \gamma \frac{\partial p^-}{\partial x}= &  +\lambda ^{p^+} p^+ -\lambda ^{p^-} p^-, \nonumber \\ p^{\pm }(x,0)= &  p_0^{\pm }(x). \end{aligned}$$

Periodic boundary conditions are set in order to minimise the influence of the domain boundaries. The turning functions $\lambda ^{p^+}$ and $\lambda ^{p^-}$ describe the rate of switching direction, from right to left and left to right, respectively. These in turn depend (non-locally) on the distribution and orientation of perceived neighbours. As stated above, we consider two fundamental interaction types: (i) attraction, to encourage cohesion, and (ii) alignment, to encourage consensus of orientation. To implement this mathematically we first assume that the turning functions increase monotonically and smoothly depend on an internal control variable $y^{p^{\pm }}$, which is a dimensionless quantity. This, as we show below, integrates the population-level travel information perceived by each group member. Specifically, guided by previous literature (Eftimie et al. 2007; Bernardi et al. 2021), we set

2 $$\begin{aligned} \lambda ^{p^\pm }=\lambda _1+\lambda _2 f(y^{p^\pm }), \quad \text { where } f(y)= 0.5+0.5 \tanh (y-y_0). \end{aligned}$$

The above form assumes the probability of changing direction has two components: a random component and a directed component that depends on the interaction with neighbours. This assumption is consistent with the biological behaviour of organisms, as also remarked in Lotka (1925) (see Eftimie 2018 for further discussion). The coefficients $\lambda _1$ and $\lambda _2$ denote the random and directed turning rate, respectively, and $y_0$ is chosen such that $f(0) \ll 1$. Next, variable $y^{p^\pm }$ is defined separately for the left and right moving populations as

3 $$\begin{aligned} y^{p^{\pm }}=\underbrace{Q_a^{p^{\pm }}}_{attraction}+\underbrace{Q_l^{p^{\pm }}}_{alignment}, \end{aligned}$$

where larger (smaller) $y^{p^{\pm }}$ correspond to a higher (lower) tendency of switching direction. Finally, the social interaction terms $Q_a^{p^\pm }$ and $Q_l^{p^\pm }$ derive from the perceived positional and directional information of the neighbours. Specifically, attraction is based on the integrated total population density, such that turning is less likely if movement is in the direction of higher population densities:

4 $$\begin{aligned} Q_a^{p^\pm } = -q_a \int _{0}^{\infty } K_a (s) \left( p(x \pm s)- p(x \mp s) \right) ds, \end{aligned}$$

where $q_a$ and $K_a(s)$ denote the attractive strength and the interaction kernel, respectively.

The alignment contribution will be different in various models, but is of the general form

5 $$\begin{aligned} Q_{l}^{p^\pm } = \pm q_{l} \int _0^{\infty } K_{l} (s) P(p^+,p^-) ds. \end{aligned}$$

where $q_l$ and $K_l(s)$ represent the alignment strength and the interaction kernel, respectively, and $P(p^+,p^-)$ defines the portion of the population that affects the alignment. For a single population model, a suitable choice would be

6 $$\begin{aligned} Q_{l}^{p^\pm } = \pm q_{l} \int _0^{\infty } K_{l} (s) [ p^{-} (x + s) + p^{-} (x - s) - (p^{+} (x + s) + p^{+} (x - s))] ds\,. \end{aligned}$$

Effectively, for individuals moving in the ($+$) direction, the above alignment contribution increases when the surrounding majority travels in the opposite (−) direction, and hence the rate of switching direction will be larger.

Finally, we note that the expressions of $Q_l$ and $Q_a$ depend on interaction kernels $K_l$ and $K_a$, which define how an interaction’s strength depends on the separation distance. Following (Eftimie et al. 2007), we take these as translated Gaussians

7 $$\begin{aligned} K_{i}(s)= \frac{1}{\sqrt{2 \pi m_{i}^2}} \exp \left( \frac{-(s-s_{i})^2}{2m_{i}^2} \right) , \quad i=a, l \quad s \in [0,\infty ), \end{aligned}$$

where $s_a$ and $s_{l}$ are half the length of the attraction and alignment ranges, respectively. This standard choice is mathematically convenient, having the advantage of infinite differentiability and bounded norms, see Eftimie (2018) for further discussion. Note that the constants $m_{i}$, $i=a, l$, are chosen such that the support of more than $98\%$ of the mass of the kernels falls inside the interval $[0, \infty )$, i.e. $m_{i}=\frac{s_{i}}{8}$, $i=a, l$. This ensures the integral defined on $[0, \infty )$ in Eq. (5) to be a reasonably accurate approximation of that defined on the whole real line. A model built on the above lines was developed and analysed in Eftimie et al. (2007). For further details, and some possible extensions, we refer to the book (Eftimie 2018).

## The *Indifferent Leaders* Model

### Formulation of the *Indifferent Leaders* Model

An extension of models as described in (2.2) to heterogeneous groups that contain both follower and leader types was considered in Bernardi et al. (2021). Here we expand on that analysis to include different leader types. In the following, we will use $u^+$ and $u^-$ to denote the right and left moving follower subpopulations, and $v^+$ and $v^-$ to denote the right and left moving leader subpopulations. Consequently, $u = u^+ + u^-$ and $v = v^+ + v^-$ denote the total follower and leader densities, respectively. We further set the total population densities, $p^+ = u^+ + v^+$, $p^- = u^- + v^-$, and $p = u+v = u^+ + v^+ + u^- + v^-$.

The *indifferent leader* model considers an extreme scenario in which leader behaviour is uninfluenced by the follower population. For example, these leaders may be entirely unrelated to followers and indifferent to their success (e.g. mariners following seabirds). To implement this in a simple manner, we introduce leaders as a compact population (assuming leaders maintain a group formation) that moves with constant speed ($\beta$) towards the right, which we subsequently refer to as the target direction. Specifically,

8 $$\begin{aligned} v^+(x,t)= &  M_{v}\exp (-0.5(x-x_0-\beta t)^2), \nonumber \\ v^-(x,t)= &  0 . \end{aligned}$$

In the above, $M_v$ denotes the maximum leader density and $x_0$ indicates the initial position of the leader group. Follower dynamics, on the other hand, are based on the system given by Eqs. (1):

9 $$\begin{aligned} \frac{\partial u^+}{\partial t} + \gamma \frac{\partial u^+}{\partial x}= &  -\lambda ^{u^+} u^+ +\lambda ^{u^-} u^-, \nonumber \\ \frac{\partial u^-}{\partial t} - \gamma \frac{\partial u^-}{\partial x}= &  +\lambda ^{u^+} u^+ -\lambda ^{u^-} u^- , \nonumber \\ u^{\pm }(x,0)= &  u_0^{\pm }(x). \end{aligned}$$

We follow the framework above by assuming the follower turning functions

10 $$\begin{aligned} \lambda ^{u^\pm }=\lambda _1+\lambda _2 [0.5+0.5 \tanh (y^{u^\pm }-y_0)], \quad \text { with } y^{u^\pm }=Q_a^{u^\pm } +Q_l^{u^\pm }. \end{aligned}$$

For the attractive contribution we assume as before an attraction according to the total population density, i.e.

11 $$\begin{aligned} Q_a^{u^\pm } = -q_a \int _{0}^{\infty } K_a (s) \left( p(x \pm s) - p(x \mp s) \right) ds \,. \end{aligned}$$

For the alignment, we adapt (6) to the form

12 $$\begin{aligned} Q_{l}^{u^\pm }= &  \pm q_{l} \int _0^{\infty } K_{l} (s) [ (u^{-} (x + s) + u^{-} (x - s)) - (u^{+} (x + s) \nonumber \\\ &  + u^{+} (x - s) + \alpha (v^+(x+s) + v^+(x-s))) ] ds\,. \end{aligned}$$

For $\alpha = 1$ the above is a straightforward generalisation of (6), but where followers now turn according to the prevailing orientation of the total (follower and leader) population. Choices of $\alpha \ne 1$, however, allow different levels of prioritisation: for example, a choice $\alpha > 1$ corresponds to a situation in which followers can discriminate between leaders and followers, and prioritise the orientation of the leaders in their alignment response.

### Dynamics of the *Indifferent Leaders* Model

Our principal objective is to assess whether the presence of the (indifferent) leader population can provide sufficient information for a group of followers to travel towards the target (here, taken to be in the “+” direction). As such, we consider initial conditions in which the groups of followers and leaders initially overlap, but the follower population is essentially unbiased with respect to its orientation. Bearing in mind the imposed leader distributions of (8), we therefore set

13 $$\begin{aligned} u^+(x,0)= &  \frac{M_{u}\exp (-0.5(x-x_0)^2)(1+r_u(x))}{2}, \end{aligned}$$

14 $$\begin{aligned} u^-(x,0)= &  \frac{M_{u}\exp (-0.5(x-x_0)^2)(1-r_u(x))}{2}, \end{aligned}$$

where $M_u$ denote the maximum follower density and $r_u(x)$ describes a random perturbation selected from a uniform distribution on $[-0.05, 0.05]$. Details of the numerical scheme are provided in Bernardi et al. (2021).

A relatively basic set of requirements for “success” at the level of the follower population would be if the following two principal features are achieved: (i) that the followers achieve group consensus for the correct direction of travel, i.e. the majority of the population moves towards the target; (ii) that the followers maintain contact with the leading group, i.e. within a distance that allows transmission of information from leaders to followers. We note that (i) in the above list would be sufficient if the target direction was fixed and the leaders travelled in a straight line path to the target, but would be insufficient otherwise: maintaining contact with the leader group would therefore be necessary for robust guidance information.

**Fig. 2:**
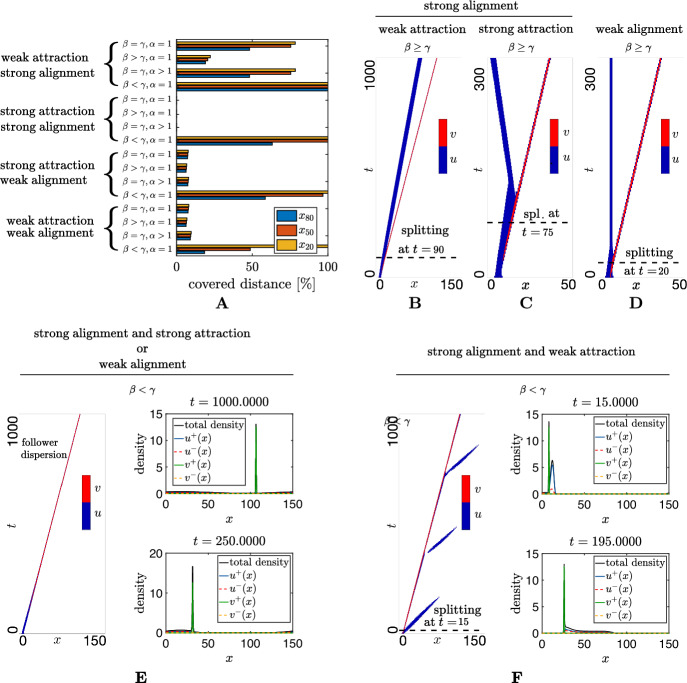
Dynamics of M1. **A** Second decile, median, and eighth decile of the follower distribution across the spatial domain at the time when the leaders have migrated 100 space units. The covered distance represents the percentage of space units traveled by the followers relative to the leaders. Four parameter regimes are investigated: weak attraction and strong alignment ($q_a=0.5, q_l=2$); strong attraction and strong alignment ($q_a=2, q_l=2$); strong attraction and weak alignment ($q_a = 2, q_l=0.1$); weak attraction and weak alignment ($q_a = 0.5, q_l=0.1$). Detachment time is evaluated when $\int _0^L u(x,t) v(x,t) dx < 0.05 \int _0^L u(x,0) v(x,0) dx$, i.e. the overlap between the follower and leader population densities in space is less than $5\%$ of its initial value. Results are shown for variations of key parameters describing speed differential between leaders and followers (i.e. $\beta \ge \gamma$, and vice versa) and leader influence on followers (i.e. $\alpha$). Specifically, we focus on: (i) $\beta =\gamma =0.1$, $\alpha =1$; (ii) $\beta =0.5$, $\gamma =0.1$, $\alpha =1$; (iii) $\gamma =\beta =0.1$, $\alpha =5$; (iv) $\beta = 0.1$, $\gamma =0.5$, $\alpha =1$. **B**–**D** Space-time evolution of densities for $\beta \ge \gamma$, under **B** weak attraction and strong alignment, **C** strong attraction and strong alignment, **D** strong attraction and weak alignment, weak attraction and weak alignment. **E**, **F** Space-time evolution of densities and leader and follower density profiles for $\beta < \gamma$, under **E** strong alignment and strong attraction, or weak alignment, **F** strong alignment and weak attraction. Other parameter values are set as $\lambda _1=0.2$, $\lambda _2=0.9$, $M_u=M_v=12.61$, and $x_0=6.5$ (Color figure online)

In Fig. 2A we describe the follower distribution across the spatial domain at the time instant when the leaders have migrated along a hundred space units. This is done by measuring the second decile, the median and the eighth decile of the follower distribution resulting from different parameter regimes. Specifically, we focus on four general parameter regimes: weak attraction and strong alignment, strong attraction and strong alignment, strong attraction and weak alignment, and weak attraction and weak alignment. Figure 2A also shows how results qualitatively change when a speed differential is introduced between leaders and followers (assuming leaders to move faster than followers, i.e., $\beta > \gamma$, or vice versa), as well as increasing the amount of leader influence on followers (i.e., $\alpha > 1$).

In the strong alignment regimes and for $\beta \ge \gamma$, the indifferent leader model can correctly align the followers on shorter timescales (see Fig. 2B, C). However, over longer time scales followers lose contact with the leader group, generating different forms of failed collective migration. Under weak attraction and strong alignment we see a splitting of the follower and leader groups, with the former travelling at a lower speed but still in the direction imparted through the brief initial contact with the leader population (see Fig. 2B). Clearly, this would be a problematic outcome if the target direction changed over time. Under strong attraction and strong alignment and $\beta \ge \gamma$, the splitting of the two populations is followed by a change of movement direction of the follower individuals (see Fig. 2C). Here, as the leaders move beyond the range of perception of followers, the followers are subject to a strong attraction towards the backward of the swarm: this leads to a rapid change of heading which is then maintained due to the strong alignment interactions. We note that (due to the imposed periodic boundary conditions) the redirected followers would eventually reconnect with the leader population as they wrap back to the right domain boundary. Of course, in most real-world scenarios, leaders would simply leave followers behind. Under weak alignment regimes and $\beta \ge \gamma$, the followers quickly lose contact with the leader group, leading to a splitting followed by followers coming to rest in the form of a stationary aggregate. Here the alignment within followers is insufficient to allow the group to polarise itself into a consensus for the group movement.

If the speed of followers exceeds that of leaders (i.e. $\beta < \gamma$) we find different examples of failed swarming. Under both strong alignment and strong attraction regimes, as well as under weak alignment regimes, we observe a progressive dispersion of the follower distribution, see Fig. 2E. In the strong alignment and weak attraction regime we observe a two-phase dynamics: first, followers split from the leaders and disperse ahead of the swarm; then, leaders “collect” the followers so that they re-accumulate at the front. Once the follower density at the front reaches a certain threshold, the follower cluster again moves at higher speeds and another dispersion takes place, see Fig. 2F. Note that for all the investigated parameter regimes we have a sufficient amount of follower to follower attraction to avoid group dispersion.

In summary, we find that indifferent leaders can guide follower migration towards the correct direction over short times, provided there is sufficient follower attraction and alignment strength and followers do not move faster than leaders. However, over longer times followers are generally unable to keep pace with the leaders, with the consequent emergence of different failure scenarios. Thus, the indifferent leader model does not appear to provide a robust guidance mechanism.

## *Observant* and *Persuadable Leaders* Models

To characterize the *observant* and *persuadable* leaders models, we introduce three means by which leaders are biased in the target direction. Building on our previous work (Bernardi et al. 2021), we assume that leaders have at least one of:

- *Orientation bias* (Obias), where leaders preferentially move in the target direction. This is modelled as a constant bias in the leaders alignment term.
- *Speed bias* (Sbias), where leaders move faster when target-directed.
- *Conspicuousness bias* (Cbias), where leaders moving towards the target are more conspicuous, e.g. visible, to followers than leaders moving away from the target. This is modelled by an appropriate weighting of the leader population within the follower alignment term.

***Model 2, the Observant leaders model***

The *Observant leader* model is the first of two models in which leaders respond to follower distributions. Consequently, we extend (1) to the following system of equations that couple follower ($u^+(x,t)$ and $u^-(x,t)$) and leader ($v^+(x,t)$ and $v^-(x,t)$) dynamics

15 $$\begin{aligned} \frac{\partial u^+}{\partial t} + \gamma \frac{\partial u^+}{\partial x}= &  -\lambda ^{u^+} u^+ +\lambda ^{u^-} u^-\, \nonumber \\ \frac{\partial u^-}{\partial t} - \gamma \frac{\partial u^-}{\partial x}= &  +\lambda ^{u^+} u^+ -\lambda ^{u^-} u^- \, \nonumber \\ \frac{\partial v^+}{\partial t} + \beta _+ \frac{\partial v^+}{\partial x}= &  -\lambda ^{v^+} v^+ +\lambda ^{v^-} v^- \, \nonumber \\ \frac{\partial v^-}{\partial t} - \beta _- \frac{\partial v^-}{\partial x}= &  +\lambda ^{v^+} v^+ -\lambda ^{v^-} v^- \, \nonumber \\ u^{\pm }(x,0)= &  u_0^{\pm }(x) \nonumber \\ v^{\pm }(x,0)= &  v_0^{\pm }(x). \end{aligned}$$

We include a potentially different speed for leaders that are moving towards the target (i.e., $\beta _+ >\beta _-$), i.e. if Sbias is in operation.

The turning functions are again given by

16 $$\begin{aligned} \lambda ^{i^\pm }=\lambda _1+\lambda _2 [0.5+0.5 \tanh (y^{i^\pm }-y_0)], \quad \text { with } y^{i^\pm }=Q_a^{i^\pm } +Q_l^{i^\pm }, \quad \text { for } i \in \{u, v\}. \end{aligned}$$

We assume the attractive interaction to be active for both leader and follower individuals: all swarm members, regardless of their follower or leader status, prefer to maintain connection with neighbours. This assumption results in the following attraction terms:

17 $$\begin{aligned} Q_a^{u^\pm } = Q_a^{v^\pm } = -q_a \int _{0}^{\infty } K_a (s) \left( u(x \pm s)+v(x \pm s)- u(x \mp s)- v(x \mp s) \right) ds\,. \end{aligned}$$

The alignment term is distinct according to each subgroup, as leaders and followers rely on different information sources. Specifically, we assume that leaders bias their movement direction according to the target, while neglecting other group members:

18 $$\begin{aligned} Q_{l}^{v^\pm } = \mp q_{l} \int _0^{\infty } K_{l} (s) \eta \, ds, \end{aligned}$$

where $\eta$ quantifies the strength of the *orientation bias* (Obias). The above states that leader alignment is uninfluenced by other swarm members, depending only on a (spatially uniform and constant) cue when the orientation bias is operating, i.e. when $\eta >0$.

Assumed to be completely uninformed regarding the destination, followers are instead taken to align according to the orientation of the neighbours,

19 $$\begin{aligned} Q_{l}^{u^\pm }= &  \pm q_{l} \int _0^{\infty } K_{l} (s) [ u^{-} (x + s) + u^{-} (x - s) + \alpha ^- (v^-(x+s) + v^-(x-s)) \nonumber \\ &  - (u^{+} (x + s) + u^{+} (x - s)) - \alpha ^+ (v^+(x+s) + v^+(x-s)) ] ds. \end{aligned}$$

Note that the target direction can potentially be favoured through $\alpha ^+ >\alpha ^-$, i.e. the Cbias that makes leaders more conspicuous when moving towards the target.

***Model 3, the Persuadable leaders model***

The *Persuadable leader* model represents the most subtle level of leader behaviour in our modelling framework, and is obtained by assuming that leader orientation incorporates both a social source of information and the impact of an environmental cue, the latter described by the parameter $\eta$ (Obias), as above (Table 1). The leader alignment term is thus the point of distinction from M2 (see Table 2) and is given by:

20 $$\begin{aligned} Q_{l}^{v^\pm }= &  \pm q_{l} \int _0^{\infty } K_{l} (s) [ u^{-} (x + s) + u^{-} (x - s) + \alpha ^- (v^-(x+s) + v^-(x-s)) \nonumber \\ &  - (u^{+} (x + s) + u^{+} (x - s)) - \alpha ^+ (v^+(x+s) + v^+(x-s)) - \eta ] ds. \end{aligned}$$

We note that in M3 the same conspicuousness bias acts equally on leader and follower alignment terms. Table 1 summarizes the model parameters varied within the manuscript. We refer to “Appendix C” for the models parameters that are kept at fixed values and set according to the previous works in Eftimie et al. (2007) and Bernardi et al. (2021).

**Table 1:** Table of parameters varied within this study

Grouping	Parameter	Description
Speed	$\beta _+$	Speed of ($+$) moving leaders
	$\beta _-$	Speed of (−) moving leaders
Attraction	$q_a$	Attraction strength
Alignment	$q_l$	Alignment strength
	$\alpha ^+$	Alignment due to ($+$) oriented leaders
	$\alpha ^-$	Alignment due to (−) oriented leaders
	$\eta$	Environmental bias perceived by leaders
Initial condition	$x_0$	Center position of aggregated initial configuration
Pop. size	$A_u$	Mean follower density
	$A_v$	Mean leader density
	$M_u$	Maximum initial follower density
	$M_v$	Maximum initial leader density
Domain	*L*	Domain length
Turning rates	$\lambda _1$	Baseline turning rate
	$\lambda _2$	Maximum turning rate

The remaining model parameters are unchanged and set according to values from previous literature, see “Appendix C”

**Table 2:** Modelling assumptions on the alignment influences for leaders and followers

Alignment sources	Followers	Observant leaders	Persuadable leaders
Direction of neighbours	✓	✗	✓
Environmental bias	✗	✓	✓

Followers do not receive information from the surrounding environment and adopt the predominant movement orientation among the neighbours. *Observant* leaders align to the perceived environmental bias. *Persuadable* leaders align to both the environmental cue and the rest of the group

## Dynamics of *Observant* and *Persuadable* Leader Models

As with the *indifferent* leader model, we examine the conditions under which each of the *observant* and *persuadable* leader models ensures successful group migration, specifically by (i) aligning the heterogeneous population to the target direction and (ii) achieving coherent movement.

### Steady States and Stability Analysis of the Non-spatial Problem

We first focus on the ability of the *observant* and *persuadable* leaders to generate consensus on group alignment towards the target, by performing a stability analysis of the non-spatial problem. Specifically, we determine the spatially homogeneous steady-states $$u^+(x,t)=u^*, u^-(x,t)=u^{**}, v^+(x,t)=v^*, v^-(x,t)=v^{**}$$, with total density $$A=A_u+A_v$$, and examine their stability. From a physical and biological perspective, the homogeneous steady state might represent an ideal scenario where individuals are aligned to the (+) or (-) direction of movement in a certain proportion, in absence of any spatial variation in the system. While this is often an abstraction of a real-world scenario, it is useful to gain an analytical handle on the system before introducing further complexities, such as spatial heterogeneity.

The steady-state set of equations for M2 reads as

21 $$\begin{aligned} h_u^{M 2}(u^*, q_l, \lambda , A_u, A_v, \alpha ^+, \alpha ^-, y_0)= &  0, \end{aligned}$$

22 $$\begin{aligned} h_v^{M 2}(v^*, q_l, \lambda , A_v, \eta , y_0)= &  0, \end{aligned}$$

where

$$\begin{aligned} h_u^{M 2}= &  -u^* \{ 1+\lambda \tanh \{2 q_l [ A_u-2u^* + \alpha ^- (A_v - v^*) - \alpha ^+ v^*]-y_0 \} \} \\ &  +(A_u-u^*) \{1 + \lambda \tanh \{ -2 q_l [ A_u-2u^* + \alpha ^- (A_v - v^*) - \alpha ^+ v^*] -y_0 \} \}, \\ h_v^{M 2}= &  - v^* [ 1+\lambda \tanh (-q_l \eta -y_0 ) ] + (A_v - v^*) [1+\lambda \tanh (q_l \eta -y_0 ) ], \end{aligned}$$

and $$\lambda = \frac{0.5 \lambda _2}{0.5 \lambda _2 + \lambda _1}$$.

From (22), we obtain a single steady-state $$(v^*,v^{**})$$ for the leader equation, where

23 $$\begin{aligned} v^*=\frac{A_v[1+\lambda \tanh ( q_l \eta - y_0)]}{2+\lambda \tanh (-q_l \eta -y_0) + \lambda \tanh (q_l \eta - y_0)}, \quad v^{**}=A_v-v^*. \end{aligned}$$

A similar set of equations is obtained for M3

24 $$\begin{aligned} &  h_u^{M 3}(u^*, q_l, \lambda , A_u, A_v, \alpha ^+, \alpha ^-, y_0) = h_u^{M 2}, \end{aligned}$$

25 $$\begin{aligned} &  h_v^{M 3}(v^*, q_l, \lambda , A_u, A_v, \alpha ^+, \alpha ^-, \eta , y_0) = 0, \end{aligned}$$

where

$$\begin{aligned} h_v^{M3}= &  - v^* \{ 1+\lambda \tanh \{ 2 q_l [ A_u-2u^* + \alpha ^- (A_v - v^*) - \alpha ^+ v^*] -q_l \eta -y_0 \} \} \\ &  + (A_v - v^*) \{1+\lambda \tanh \{ -2q_l [A_u-2u^* +\alpha ^- (A_v - v^*) - \alpha ^+ v^*] +q_l \eta -y_0 \} \}. \end{aligned}$$

If there is no alignment, i.e. $$q_l=0$$, for both M2 and M3 we find the unaligned steady-state with both populations equally distributed in the (±) direction of movement, i.e. $$(u^*, u^{**}, v^*, v^{**})=\left( \frac{A_u}{2}, \frac{A_u}{2}, \frac{A_v}{2}, \frac{A_v}{2} \right) $$. If $$q_l \ne 0$$, the steady-state expression is more intricate and to gain insight we focus on a few particular scenarios. Note that Sbias, i.e. $$\beta _+ \ne \beta _-$$, has no effect on the steady-state equations: we thus consider only how the other leader biases, i.e. Obias and Cbias, impact on steady states.

The unbiased case is obtained when $$\eta =0$$, $$\alpha ^+=\alpha ^-=1$$. This reflects an absence of any leader source of movement information and in this case leaders are indistinguishable from followers. In M2, we find $$(v^*, v^{**})= (\frac{A_v}{2}, \frac{A_v}{2})$$ and the follower steady-state equation reduces to

26 $$\begin{aligned} h_u^{M 2}=-u^* \{ 1+\lambda \tanh \{2 q_l [ A_u-2u^*]-y_0 \} \} +(A_u-u^*) \{1 + \lambda \tanh \{ -2 q_l [ A_u-2u^* ] -y_0 \} \}. \end{aligned}$$

**Fig. 3:**
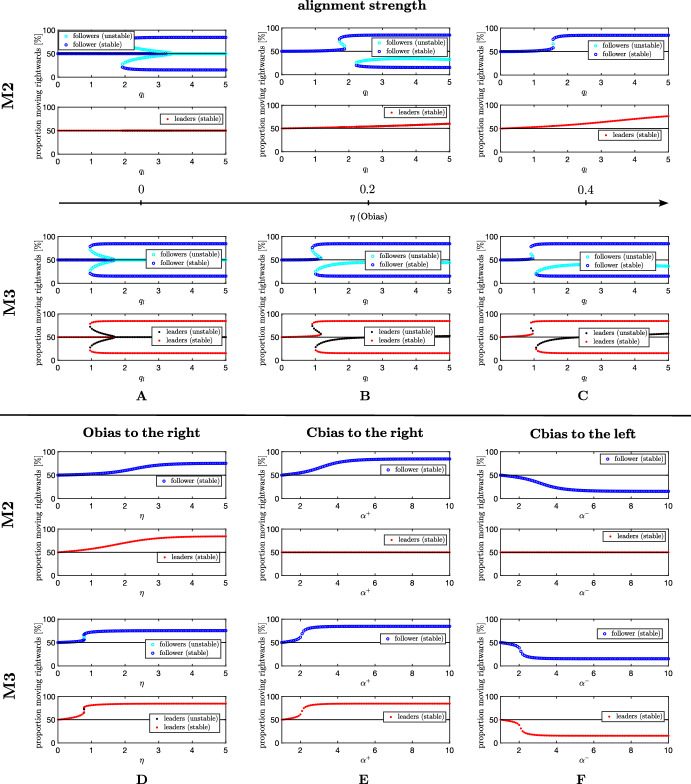
M2 and M3, Proportion of right-moving populations at steady state(s). **A**–**C** Effect of $q_l$ on position and number of equilibrium points, for $\eta =0$, $\eta =0.2$, $\eta =0.4$. **D** Effect of information level of leaders $\eta$ on position and number of equilibrium points, for $q_l=0.8$. **E**, **F** Effect of the influence of right-moving (left-moving) leaders on followers $\alpha ^+$ ($\alpha ^-$) on position and number of equilibrium points, for $\alpha ^-$ = 1 ($\alpha ^+$ = 1) and $q_l=0.5$. Other parameter values fixed at $A_u = A_v = 1$, $\lambda _1 = 0.8$, and $\lambda _2 = 3.6$ (Color figure online)

In the context of a homogeneous population, the latter equation has been widely studied in Eftimie et al. (2007). We restrict here to noting that the number of follower steady-states shifts between one, three and five, depending on the alignment strength $q_l$ (see Fig. 3A, top row). A sufficiently high alignment strength results in the stable follower steady state shifted to one in which a preferential direction of movement is selected (consensus). However, without any bias, consensus is symmetric with respect to both left (−) and right ($+$) directions. In M3, under the same unbiased scenario, we find $h_u^{M 3}=h_v^{M 3}=h^{M 3}$: leader and follower steady-states coincide (see Fig. 3A, bottom row) and become the solution of the following

27 $$\begin{aligned} h^{M 3}= &  -u^* \{ 1+\lambda \tanh \{2 q_l [ A_u-2u^* + A_v - 2v^*)]-y_0 \} \} \nonumber \\ &  +(A_u-u^*) \{1 + \lambda \tanh \{ -2 q_l [ A_u-2u^* + A_v - 2v^*] -y_0 \} \}. \end{aligned}$$

The symmetry of the system obtained in the unbiased scenario is compromised when $\eta > 0$ (see Fig. 3B, C). The upper branches (corresponding to the target direction) are more likely to be selected, while the lower ones are shifted to the right and completely disappear for larger values of $\eta$. This is obtained for both M2 and M3; however, while consensus can be achieved in M3 at lower values of $q_l$ compared to M2, M3 requires much higher values of $\eta$ to make the lower branch disappear. This reflects that the orientation of persuadable leaders for M3 also stems from the orientation of followers, which, in turn, contribute to the determination of leader direction of movement. This process helps in reinforcing the target direction when $q_l$ is limited. However, higher values of $q_l$ amplify the information coming from the neighbours and larger values of $\eta$ are required in M3 to overcome the social influence. For M2, the single leader steady-state is also shifted towards the (+) direction when $\eta >0$.

We also investigate the effect of increasing Obias strength when Cbias is inactive ($$\eta \ne 0, \alpha ^+=\alpha ^-=1$$). For both M2 and M3, if the alignment is not enough to provide a preferential alignment of the population, a small amount of environmental bias for leaders, $$\eta \ne 0$$, provides consensus. This is the situation depicted in Fig. 3D, where leaders and followers are equally distributed in left and right moving orientation when $$\eta =0$$. Under the extreme Obias scenario, i.e. $$\eta \rightarrow \infty $$, both *observant* and *persuadable* leaders in M2 and M3 favour the (+) direction, i.e. $$(v^*, v^{**})=(\frac{A_v(1+\lambda )}{2}, \frac{A_v(1-\lambda )}{2})$$, and the follower steady-state equation reduces to

28 $$\begin{aligned} h_u^{M2}=h_u^{M3}= &  -u^* \left\{ 1+\lambda \tanh \left\{ 2 q_l \left[ A_u-2 u^*- A_v \lambda \right] -y_0 \right\} \right\} \nonumber \\ &  +(A_u-u^*) \left\{ 1 + \lambda \tanh \left\{ -2 q_l \left[ A_u-2 u^*- A_v \lambda \right] -y_0 \right\} \right\} . \end{aligned}$$

The symmetric structure of Eqs. (26) and (27) is therefore lost due to the quantity $A_v \lambda$, which acts to favour a shift of the steady-states towards the biased direction. Note also that in M3 a lower value of $\eta$ with respect to M2 is sufficient to reach consensus. Again, this is due to the positive feedback between leaders and followers which favours the transfer of the target direction of movement, provided a limited alignment strength $q_l$ ($q_l = 0.8$ in Fig. 3D).

We then turn to examine the effect of Cbias when Obias is inactive ($\alpha ^+ / \alpha ^-, \alpha ^- / \alpha ^+ > 0$, $\eta =0$). If the influence of right-moving (left-moving) leaders is sufficiently large the follower population will largely adopt a rightward (leftward) orientation movement, even in absence of the environmental bias, see Fig. 3E, F. Indeed, for both M2 and M3, the follower steady-states are shifted towards the (+) or (-) direction, respectively. The same shift is observed for leader steady-state in M3 while in M2 leaders settle into the unaligned one (see Fig. 3E, F). Consistently, we remark that $h_v^{M2}=0$ is not affected by Cbias. Even if the leaders are equally distributed in orientation, a sufficiently high conspicuousness of their target-directed proportion provides consensus. This situation biologically occurs in honeybee swarming behaviour, where leaders are observed to fly back and forth within the swarming cloud with noticeable fast streaks when target directed. For $\alpha ^+ / \alpha ^- \rightarrow \infty$, we find

$$\begin{aligned} &  (u^*, u^{**},v^*, v^{**})=\left( \frac{A_u(1+\lambda )}{2}, \frac{A_u(1-\lambda )}{2},\frac{A_v}{2}, \frac{A_v}{2}\right) \text { for M2 and }\\ &  (u^*, u^{**},v^*, v^{**})=\left( \frac{A_u(1+\lambda )}{2}, \frac{A_u(1-\lambda )}{2},\frac{A_v(1+\lambda )}{2}, \frac{A_v(1-\lambda )}{2}\right) \text { for M3. } \end{aligned}$$

To summarise, bifurcation diagrams in Fig. 3 corroborate these findings and allows us to highlight the differences between the leader behaviour described in M2 and M3 in terms of the key parameters underlying the information transfer from leaders to followers, i.e. the alignment strength $q_l$, the environmental bias $\eta$, and the influence of target-directed leaders on followers quantified by the ratio $\frac{\alpha ^+}{\alpha ^-}$. Colours refers to the stability properties of the steady states (details on stability analysis of the time-only problem are included in the “Appendix A”). The greater follower influence on the *persuadable* leader behaviour described in M3 generally facilitate the coordination process: a lower value of $q_l$, $\eta$, and $\alpha ^+ / \alpha ^-$ with respect to M2 is indeed sufficient to reach consensus. On the other hand, this positive feedback may lead the group to choose the wrong direction when alignment is too high (see Fig. 3A–C (bottom row)). These results thus suggest that M3 expands the parameter region where consensus is obtained, provided an optimal amount of alignment is set. Moreover, we have shown the effect of the crucial parameters on the configuration of the state states. Specifically, $q_l$ generates different solutions structured in orientation while the bias parameters, i.e. $\eta$ and $\alpha ^\pm$, shift them to one preferential direction of orientation.

***Effect of conflicting information on leader guidance***

**Fig. 4:**
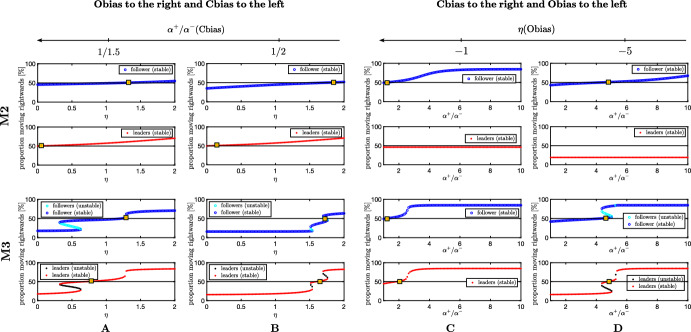
M2 and M3, Proportion of right-moving populations at steady state(s). **A**, **B** Effect of $\eta >0$ on position and number of equilibrium points: **A** $\frac{\alpha ^+}{\alpha ^-} = \frac{1}{1.5}$, **B** $\frac{\alpha ^+}{\alpha ^-} = 0.5$, with $q_l=0.8$. **C**, **D** Effect of $\frac{\alpha ^+}{\alpha ^-} > 0$ on position and number of equilibrium points: **C** for $\eta =-1$, **D** $\eta =-5$, with $q_l=0.5$. Other parameter values fixed at $A_u = A_v = 1$, $\lambda _1 = 0.8$, and $\lambda _2 = 3.6$. Note that the yellow square with a red star indicates the position where the steady state is symmetric with respect to the proportion of rightward v leftward oriented individuals (Color figure online)

Now we discuss the effect of conflicting information (i.e. $\eta < 0$ and $\frac{\alpha ^+}{\alpha ^-} > 1$, and vice versa) on the position and number of steady-states, see Fig. 4. When leaders are (even slightly) more conspicuous when moving to the left (i.e. $\frac{\alpha ^+}{\alpha ^-}<1$), the effect of an environmental bias to the right, i.e. $\eta >0$, is consequently reduced in both M2 and M3, and larger values of $\eta$ are needed to have the (+) direction favoured, see Fig. 4A, B. The opposite situation is depicted in Fig. 4C, D. In presence of a negative environmental bias of leaders, i.e. $\eta <0$, if the conspicuousness of (+)-oriented leaders is sufficiently large, a consensus may be reached anyway. This occurs also in M2 where leader population settle into a predominantly (-)-oriented steady-state, regardless of the Cbias strength. Generally, the stronger the Obias (Cbias) to the left, the larger the influence of right-moving leaders (right environmental leader bias) needs to be in order to reach a consensus where right orientation is favoured. Moreover, for a fixed rightwards Obias (Cbias) strength, the $\alpha ^+ / \alpha ^-$ ($\eta$) threshold value required to generate consensus is higher in M2 w.r.t. M3, confirming the effect of reinforcement of information for *persuadable* leaders (M3). Interestingly, hysteresis loops are observed in M3 by varying $\eta$ (Fig. 4A, B) and $\alpha ^+$ (Fig. 4D), suggesting that decreasing the bias parameter from a state of consensus may not lead to the group realigning to its previous state, e.g. potentially due to learned behaviour.

### Numerical Simulations

**Fig. 5:**
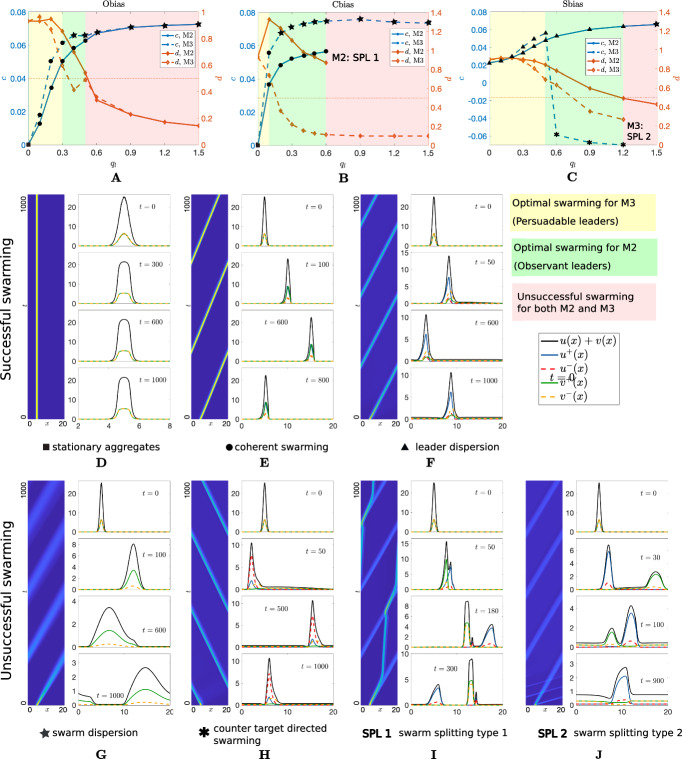
M2 and M3, Effect of Obias, Sbias and Cbias leader strategies on the swarm dynamics as $q_l$ increases under low attraction regimes and clustered initial configuration. **A**–**C** swarm dynamics is evaluated in terms of the speed (in blue) and the cohesion index (in orange) of the follower population. We highlight in yellow the $q_l$ values for which optimal swarming is obtained for M3, in green those for which optimal swarming is obtained for M2. Red regions denote unsuccessful swarming for both M2 and M3. Numerical simulations are obtained for **A** $\eta =10$, $\alpha ^\pm =1$, $\beta _\pm =0.1$, **B** $\beta _+/\beta _-=0.5/0.1$, $\eta =1$, $\alpha ^\pm =1$, **C** $\alpha ^+/\alpha ^-=5/1$, $\beta _\pm =0.1$, $\eta =1$. **D**–**F** examples of successful swarming patterns displayed by M2 and M3 (see correspondence with panels in the first row). **G**–**J** examples of unsuccessful swarming patterns displayed by M2 and M3 (see correspondence with panels in the first row). Other parameter values are set as $q_a=0.5$, $\lambda _1=0.2$, $\lambda _2=0.9$, $M_u=12.61$, $M_v=12.61$, and $x_0=5$ (Color figure online)

The steady state and stability analyses of the non-spatial problem have revealed insight into the alignment process provided by the the *observant* and *persuadable* leader models. We now incorporate the spatial variable into the problem to investigate their ability to generate a spatially coherent group migration. In this regard, a numerical approach is necessary to explore the complex dynamics in space. In Fig. 5 we test the efficiency of different guidance strategies (Obias, Cbias and Sbias) when adopted by observant and persuadable leaders, respectively described by M2 and M3. Given that the point of distinction between M2 and M3 lies in the leaders’ alignment terms, we examine how the resulting collective behaviour changes with variations in the alignment strength. The other parameter values selected for the numerical realizations are chosen such that the linear stability analysis of the uniform solution predicts Turing pattern formation (the emerging aggregates are predicted to attain a spatially grouped configuration) in the unbiased case, i.e. when no leader bias is in operation, see “Appendix B”. In this respect, an attraction strength $q_a$ above a certain threshold turns out to play a key role in aggregating the population, which is of particular interest in the context of collective movement. Specifically, sufficiently strong attraction allows the populations to form and maintain a grouped form.

When introducing the effect of the three different leader strategies, we will refer to successful swarming dynamics if the follower group (i) moves in the direction of the target from their origin and (ii) maintains a compact group (i.e., follower individuals are not lost). Accordingly, we describe the resulting dynamics in terms of two relevant quantities. First, we look at the mean speed of the follower aggregation over the whole observation time. Secondly, we track a “cohesion index” measured as $d=\frac{d_0}{d_{end }}$, where $d_0$ and $d_{end }$ respectively denote the spatial extension $d=x_{80}-x_{20}$ of the follower aggregation measured at the initial time instant and at the end of the observation time, being $x_{20}$ and $x_{80}$ the second and the eighth deciles of the follower distribution. In this respect, $d \approx 1$ will reflect that a good coherence is maintained during the follower migration; conversely, $d \ll 1$ reflects follower dispersion.

The results show a clear trade-off: as the alignment strength increases, the speed of the groups increases while the cohesion index decreases for both M2 and M3. Furthermore, for lower values of $q_l$ all guidance strategies under M3 generates higher speed for the migrating swarm compared to M2 (see Fig. 5, first row, yellow regions). For higher values of $q_l$, M2 instead offers a better swarming, as for M3 we observe a drop in the cohesion index under the action of Obias and Cbias, while Sbias generates a counter-directed swarming (see Fig. 5, first row, green regions). Even higher values of $q_l$ lead to unsuccessful swarming behaviour for both M2 and M3, including swarm dispersion, counter target directed swarming and two types of swarm splitting (with the leaders leaving the followers behind, and vice versa).

M2 and M3 thus generate a variety of successful and unsuccessful swarming dynamics and we discuss some of the swarming scenarios below. Due to being incorporated within the alignment term, both Obias and Cbias can lead to stationary aggregates when there is no alignment, i.e. $q_l=0$ suppresses Obias and Cbias action and the swarm self-organizes into a compact cluster through attraction, see Fig. 5A, Fig. 5B, D. If $q_l=0$, Sbias can however provide a coherent (though slow) follower migration towards the target, while leaders gradually disperse, see Fig. 5C, F. Indeed, leaders with a greater speed towards the target are able to broadcast travelling information to the followers through attraction but are not able to self maintain a compact cluster, due to being both attracted both to slower follower and other faster leader individuals. When $q_l > 0$, the action of Obias and Cbias in M2 and M3 turns out to be the best guidance process with both leader and follower populations compactly moving to the destination, provided the alignment strength is not excessively strong, see Fig. 5A, B (green and yellow regions), and Fig. 5E. When $q_l$ passes above a certain threshold, under M3 both Obias and Cbias can lead to dispersion of both leader and follower populations, see Fig. 5A, Fig. 5B (red regions) and Fig. 5G. The same result is observed under M2 for an Obias strategy, while Cbias generates swarm splitting of type I. In this case, leaders evolve to a stationary configuration and the followers split away from them in the direction of the target, being favoured from the initial propulsive thrust received from leaders, see Fig. 5B (red region) and Fig. 5I. Similarly, if alignment is too high then Sbias provides a failure swarming scenario with swarm dispersion under M2, see Fig. 5C (red region), and swarm splitting of type II under M3, with faster leaders leaving the followers behind, see Fig. 5C (red region) and Fig. 5J.

Under a higher attraction regime, we find a successful swarming dynamics for all the investigated $q_l$ values (see Fig. 8, “Appendix D”). In particular, increasing the attraction strength expands the parameter regions that represent best swarming for M3 (yellow regions) and M2 (green regions), respectively. This highlights the positive effect of attraction between group members on the guidance success.

To summarise, our numerical study suggests that when the alignment connection between all group members is limited, greater follower influence on leaders is beneficial for successful guidance. On the contrary, if the alignment connection is strong enough, a greater follower influence on leaders can be detrimental and may result in unsuccessful swarming.

## Discussion

Follower-leader heterogeneity often underlies the success of collective migration processes and occurs at different spatial and temporal scales, from cellular to animal systems (Qin et al. 2021; Jolles et al. 2017; Pettit et al. 2015). Such systems are characterized by the presence of knowledgeable and/or experienced leaders, who guide the movement direction for the naive followers. However, this clear-cut division overlooks a broader range of heterogeneity that can characterize the composition of the group. In this respect, we have investigated different degrees of leadership, ranging from highly certain leaders to uncertain leaders, extending the recent non-local hyperbolic model for follower-leader systems proposed in Bernardi et al. (2021). Specifically, we have considered three degrees of leadership: indifferent leaders, who have no response to other group members; observant leaders, who show an attraction response to the neighbours, and persuadable leaders, who have both an attraction and an alignment response towards neighbours. Focusing on the ability of the resulting leader behaviour to (i) broadcast the target direction to the follower and (ii) keep the migrating group cohesive without dispersion, we have found that indifferent leaders do not provide successful guidance for long times, leading to group splitting or follower dispersion. Thus, a kind of social connection from leaders to followers seems to be required for a successful guidance. The importance of balancing goal-oriented and social-oriented behaviours for leaders is further supported by the experimental observations in Ioannou et al. (2015), where trained-to-target fish that exhibit high goal orientation—characterized by faster and straighter paths to target—and low social behaviour are likely to quickly reach the preferred target but tend to leave untrained fish behind, resulting in a failure to transmit their preference to others.

Both observant and persuadable leaders instead provide robust guidance, showing that the best leader strategy depends on the alignment connection between the migrating individuals: a greater follower influence on leaders generally helps the guidance process when the alignment connection between individuals is limited; otherwise, it may generate an excess amount of directional information and lead to unsuccessful swarming dynamics. This result emerges both analytically, from the steady-state of the non-spatial problem and its stability properties, and numerically from simulations under moderate parameter variations. Moreover, it appears robust to variations in the three modelled mechanisms through which leaders can be biased towards the target direction: orientation (Obias), increased speed (Sbias), and conspicuousness (Cbias).

Although the model is not designed to describe any specific system, but rather to provide general insights, examples from animal collective movement substantiate our findings. Observant leadership, for instance, is evident in migrating schools of fish and swarming honeybees. Specifically, target-trained fish demonstrate both individual knowledge of the target (to reach the food reward) and a strong tendency to maintain group cohesion (Miller et al. 2013). Informed scout bees perform high-speed flights through the migrating swarm while maintaining contact with the majority of uninformed bees (Schultz et al. 2008). Similarly, persuadable leader behaviour, which pools partial estimates of the homeward route with social information, is key to the effective migration of pigeon flocks (Herbert-Read 2015; Pettit et al. 2015).

The study offers deep insights into different leader behaviours, yet some simplifying assumptions have been required by the complexity of the modelling framework. We note that the proposed modelling framework is one-dimensional, despite the fact that the majority of collective dynamics in cell and animal communities occur in higher spatial dimensions, i.e. 2D or 3D. While some 2D and 3D patterns might approximate to a similar 1D structure as observed in this study (e.g., stationary aggregates, coherent swarming, swarm dispersion, swarm splitting), we must remain mindful that a much wider variety of patterns could emerge in higher dimensions (see also Eftimie 2018). While desirable, the drawback of the higher-dimensional framework lies in the significant increase in model complexity: possible velocities extend from two ($+$ or −) to all vectors of an $n-$dimensional ball. Then, a turning kernel $T({\textbf{v}}, \mathbf {v'})$ would describe the probability of a velocity to change from ${\textbf{v}}$ to $\mathbf {v'}$, in response to social interactions. In this respect, the extension of the hyperbolic model proposed in Eftimie et al. (2007) to two dimensions has been formulated and investigated in Fetecau (2011) for homogeneous populations, and in Fetecau and Meskas (2013) for heterogeneous populations (in the context of predator–prey dynamics). Another modelling framework that can be used to study the role of heterogeneity on collective movement in multiple spatial dimensions lies in the non-local advection–diffusion model reviewed in Painter et al. (2024b), which is more amenable to analysis and (for example) has recently been devoted to investigating chase-and-run dynamics (Painter et al. 2024a).

We also note that our investigation has restricted to the choice of periodic boundary conditions, which mimics a circular arena-like domain and allows any influence of boundaries to be minimised (Eftimie 2018). This choice is also dictated by mathematical convenience, as dealing with non-local models requires one to not only define the boundary conditions, but also how the non-local terms behave near boundaries (the integrals involved in the non-local terms are here simply wrapped around the domain). This issue has been explored in Hillen and Buttenschön (2020) for another form of non-local model, where the authors derive different types of boundary conditions for non-local models. Other boundary conditions relevant for models describing collective organization and movement could include reflecting (Neumann) boundary conditions to describe walled domains, or zero (Dirichlet) boundary conditions to describe domains surrounded by regions which do not sustain the survival of the population. We refer to Eftimie (2018) for further discussion.

Since one of the key factors affecting the individual ability to gain directional information is the surroundings (e.g. weather condition, physical landscape), the environmental conditions under which the migration occurs may be crucial in determining which is the best leader behaviour to be adopted—specifically, the extent to which the leader’s behaviour gain benefit in being influenced by the rest of the group. In this regard, the influence of the environment in shaping individual and/or behavioural heterogeneity has been observed in both animal and cellular systems (Jolles et al. 2020; McLennan et al. 2012). An illustrative example is neural crest cell migration during embryogenesis. Studies here have shown the importance of the chemoattractant VEGF, not only in providing the direction of invasion but also in determining follower or leader status: cells exposed to higher VEGF gradients become leader cells, while those in lower gradients become followers (McLennan et al. 2015). In this respect, it would be interesting to explicitly incorporate environmental effects into the model, relaxing the assumption of fixed individual behaviour and instead making it depend on an environmental variable (e.g., quantifying the effect of a specific chemical factor, weather condition, or predation risk). Developing the model in this direction would require additional switching terms to account for transitions between different leader behaviours, and potentially between follower and leader modes as well. In this respect, transient leadership has already been investigated using kinetic models, with the leader-follower status described as either a discrete trait (Albi and Ferrarese 2024) or a continuous trait (Cristiani et al. 2025).

**Fig. 6:**
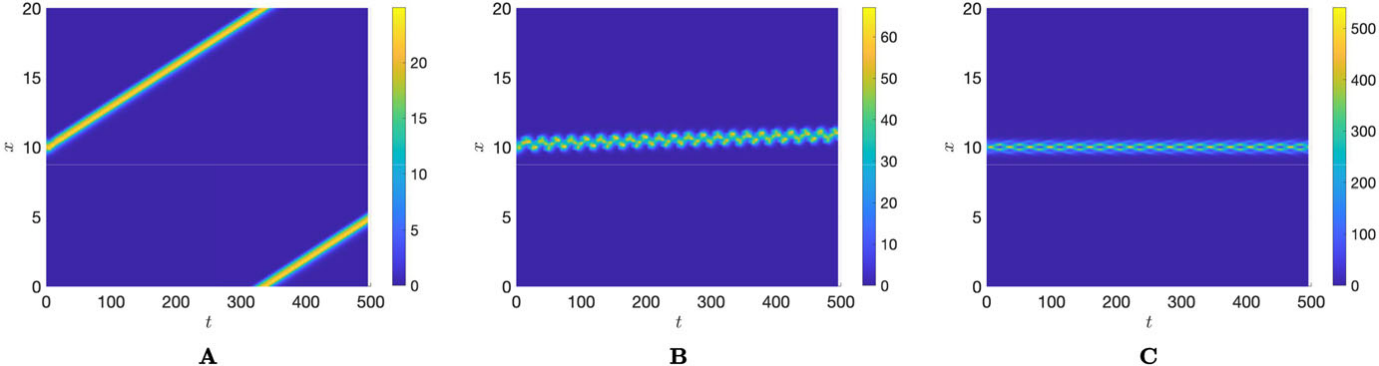
Effect of increasing the total follower population on the swarming dynamics described by M3. **A** Moving pattern obtained for $M_u=10$, **B** pulsating-moving pattern obtained for $M_u=30$, **C** pulsating-stationary pattern obtained for $M_u=200$, leaving unchanged $M_v=10$. Other parameters are set as $\gamma =\beta _{\pm }=0.1, q_a=2, q_l=0.5, \eta =0.5$, and $\alpha ^\pm =1$ (Color figure online)

**Fig. 8:**
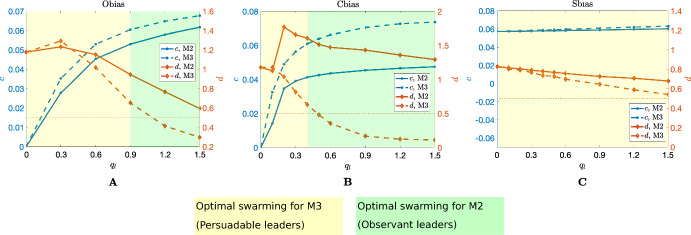
M2 and M3, Effect of Obias, Sbias and Cbias leader strategies on the swarm dynamics as $q_l$ increases under high attraction regime and clustered initial configuration. Numerical simulations are obtained for **A** $\eta =10$, $\alpha ^\pm =1$, $\beta _\pm =0.1$, **B** $\beta _+/\beta _-=0.5/0.1$, $\eta =1$, $\alpha ^\pm =1$, **C** $\alpha ^+/\alpha ^-=5/1$, $\beta _\pm =0.1$, $\eta =1$. Swarm dynamics is evaluated in terms of the speed (in blue) and the cohesion index (in orange) of the follower population. In yellow and green, we highlight the $q_l$ values for which optimal swarming is obtained for M3 and M2, respectively. Other parameter values are set as $q_a=2$, $\lambda _1=0.2$, $\lambda _2=0.9$, $M_u=12.61$, $M_v=12.61$, and $x_0=5$ (Color figure online)

We also note that this study has focused on fixed total densities of leaders and followers, which have been set to be equal. In this regard, preliminary numerical results suggest that increasing the follower population size leads to the emergence of pulsating-type patterns where the target-directed group migration provided by the leaders (specifically, Obias) can be lost (see Fig. 6). A more in-depth study of these dynamics would be valuable (e.g., to explore how large the follower population can be while still being successfully led by a given leader population), though it falls outside the scope of this work. We also note that follower and leader speeds, i.e. $\beta _\pm$ and $\gamma$, are kept fixed throughout the study, while modulation of the direction of movement is taken into account only by turning rates. This does represent a limitation of our model, as including the possibility of directly modulating individual speeds would be both a reasonable assumption and an interesting point for further investigation. Another relevant aspect is that we have kept the interaction ranges of the follower and leader populations fixed and equal. Recent work in Painter et al. (2024a) has shown the impact of variations in non-local interaction ranges on the emergence of another form of group heterogeneity, namely chase-and-run dynamics. In chase-and-run systems, individuals of the first population move towards those of the second population, which, in turn, move away. Similar to follower-leader systems, these dynamics occur in populations of non-locally interacting cells or animals. In Painter et al. (2024a), the authors show that chase-and-run dynamics are strongly influenced by non-local interaction terms and occur when the sensing range of the chaser exceeds that of the runner. Building on this, it would be interesting to investigate whether varying the sensing ranges of leader and follower populations could lead to similar results in the modelling framework used here.

The model could also be adapted to study the co-presence of competing leaders behaviours pointing to different directions in a decision-making perspective. In this respect, the dynamics emerging from the presence of two subsets of informed individuals, each having its own directional preference, is studied using a discrete approach in Couzin et al. (2005).

Furthermore, a promising direction would be the integration of data-such as measurements of speed, movement direction, and tracking data from migrating cell or animal populations-into the proposed model, in order to tailor it and gain insights into specific systems. Despite the rapidly increasing availability of such data thanks to modern technologies, incorporating them into hyperbolic models remains an open research challenge, as also discussed in Eftimie (2018).

In conclusion, these models offer a solid step towards a broader investigation on the leader-follower heterogeneity underlying a variety of biological and ecological processes, offering insights on the individual mechanisms that may drive biological dynamics observed at the population level.

## Figures and Tables

**Fig. 7:**
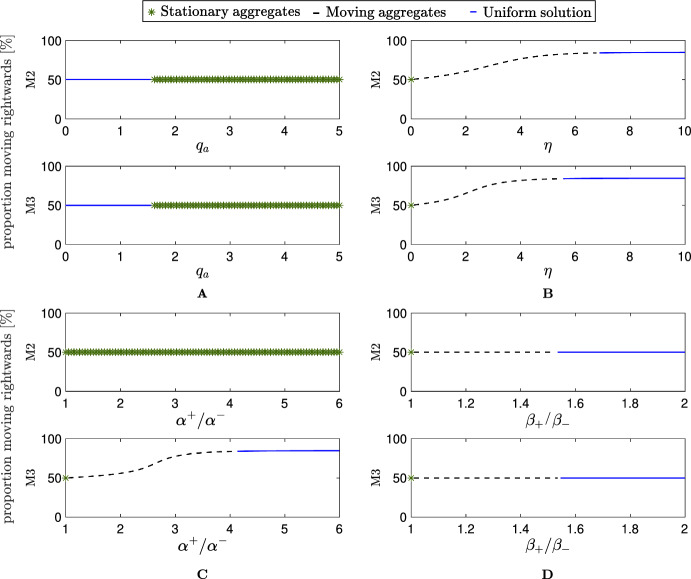
M2 and M3 leader-only sub-models, Proportion of right-moving populations at steady state. Effect of **A** attraction strength $q_a$, **B** $\eta$ (Obias strength), **C** $\alpha ^+/\alpha ^-$ (Cbias strength) and **D** $\beta _+/\beta _-$ (Sbias strength) on position and linear stability properties of steady states. Other parameter values: $q_l=0.5$, $q_a=2$ in (**B**–**D**), $\lambda _1=0.2$, and $\lambda _2=0.9$ (Color figure online)

## Data Availability

The manuscript has no associated data.

## Appendix A: Stability Analysis of the Non-spatial Problem

The time-only problem of models M2 and M3 presented in Eq. (15) reduces to

A1 $$\begin{aligned} \frac{\partial u^+}{\partial t}= &  -\lambda ^{u^+} u^+ +\lambda ^{u^-} u^-\,= f(u^+, u^-, v^+, v^-), \nonumber \\ \frac{\partial u^-}{\partial t}= &  +\lambda ^{u^+} u^+ -\lambda ^{u^-} u^- \,= g(u^+, u^-, v^+, v^-), \nonumber \\ \frac{\partial v^+}{\partial t}= &  -\lambda ^{v^+} v^+ +\lambda ^{v^-} v^- \,= p(u^+, u^-, v^+, v^-), \nonumber \\ \frac{\partial v^-}{\partial t}= &  +\lambda ^{v^+} v^+ -\lambda ^{v^-} v^- \, = q(u^+, u^-, v^+, v^-). \end{aligned}$$

To assess the stability properties of the steady states, we evaluate the Jacobian matrix for the system (A1) at a generic point:

A2 $$\begin{aligned} J=\begin{pmatrix} \frac{\partial f}{\partial u^+} & \frac{\partial f}{\partial u^-} & \frac{\partial f}{\partial v^+} & \frac{\partial f}{\partial v^-} \\ \frac{\partial g}{\partial u^+} & \frac{\partial g}{\partial u^-} & \frac{\partial g}{\partial v^+} & \frac{\partial g}{\partial v^-} \\ \frac{\partial p}{\partial u^+} & \frac{\partial p}{\partial u^-} & \frac{\partial p}{\partial v^+} & \frac{\partial p}{\partial v^-} \\ \frac{\partial q}{\partial u^+} & \frac{\partial q}{\partial u^-} & \frac{\partial q}{\partial v^+} & \frac{\partial q}{\partial v^-} \end{pmatrix}. \end{aligned}$$

Straightforward algebra leads to the following expressions for $\frac{\partial i}{\partial j^\pm }$, for $i \in \{f, g, p, q\}, j \in \{u, v\}$:

$$\begin{aligned} \frac{\partial f}{\partial u^+}= &  -\frac{\partial g}{\partial u^+} =-\frac{\partial \lambda ^{u^+}}{\partial u^+} u^+ - \lambda ^{u^+} + \frac{\partial \lambda ^{u^-}}{\partial u^+} u^-, \\ \frac{\partial f}{\partial u^-}= &  -\frac{\partial g}{\partial u^-} =-\frac{\partial \lambda ^{u^+}}{\partial u^-} u^+ + \frac{\partial \lambda ^{u^-}}{\partial u^-} u^- + \lambda ^{u^-}, \\ \frac{\partial f}{\partial v^+}= &  -\frac{\partial g}{\partial v^+} =-\frac{\partial \lambda ^{u^+}}{\partial v^+} u^+ + \frac{\partial \lambda ^{u^-}}{\partial v^+} u^-, \\ \frac{\partial f}{\partial v^-}= &  -\frac{\partial g}{\partial v^-} = -\frac{\partial \lambda ^{u^+}}{\partial v^-} u^+ + \frac{\partial \lambda ^{u^-}}{\partial v^-} u^-, \\ \frac{\partial p}{\partial u^+}= &  -\frac{\partial q}{\partial u^+} =-\frac{\partial \lambda ^{v^+}}{\partial u^+} v^+ + \frac{\partial \lambda ^{v^-}}{\partial u^+} v^-, \\ \frac{\partial p}{\partial u^-}= &  -\frac{\partial q}{\partial u^-} =-\frac{\partial \lambda ^{v^+}}{\partial u^-} v^+ + \frac{\partial \lambda ^{v^-}}{\partial u^-} v^-, \\ \frac{\partial p}{\partial v^+}= &  -\frac{\partial q}{\partial v^+} =-\frac{\partial \lambda ^{v^+}}{\partial v^+} v^+ -\lambda ^{v^+}+ \frac{\partial \lambda ^{v^-}}{\partial v^+} v^-, \\ \frac{\partial p}{\partial v^-}= &  -\frac{\partial q}{\partial v^-} = -\frac{\partial \lambda ^{v^+}}{\partial v^-} v^+ + \frac{\partial \lambda ^{v^-}}{\partial v^-} v^- +\lambda ^{v^-}. \\ \end{aligned}$$

In the above, for the *Observant* leader model (M2) we have

$$\begin{aligned} \frac{\partial \lambda ^{u^+}}{\partial u^\pm }= &  \mp \lambda _2 \, q_l \, [1-\tanh ^2(y^{u^+}_{M2}-y_0)], \\ \frac{\partial \lambda ^{u^+}}{\partial v^\pm }= &  \mp \lambda _2 \, q_l \, \alpha ^{\pm } [1-\tanh ^2(y^{u^+}_{M2}-y_0)], \\ \frac{\partial \lambda ^{u^-}}{\partial u^\pm }= &  \pm \lambda _2 \, q_l \, [1-\tanh ^2(y^{u^-}_{M2}-y_0)], \\ \frac{\partial \lambda ^{u^-}}{\partial v^\pm }= &  \pm \lambda _2 \, q_l \, \alpha ^{\pm } [1-\tanh ^2(y^{u^-}_{M2}-y_0)], \\ \frac{\partial \lambda ^{v^+}}{\partial u^\pm }= &  \frac{\partial \lambda ^{v^+}}{\partial v^\pm }= 0, \\ \frac{\partial \lambda ^{v^-}}{\partial u^\pm }= &  \frac{\partial \lambda ^{v^-}}{\partial v^\pm }= 0, \\ \end{aligned}$$

where the perceived signal function is given as:

$$\begin{aligned} y^{u^{\pm }}_{M2}= &  Q_l^{u^\pm }= \pm 2 \, q_l ( u^- + \alpha ^- v^- - u^+ -\alpha ^+ v^+). \end{aligned}$$

For the *Influenced* leader model (M3) we have

$$\begin{aligned} \frac{\partial \lambda ^{u^+}}{\partial u^\pm }= &  \mp \lambda _2 \, q_l \, [1-\tanh ^2(y^{u^+}_{M3}-y_0)], \\ \frac{\partial \lambda ^{u^+}}{\partial v^\pm }= &  \mp \lambda _2 \, q_l \, \alpha ^{\pm } [1-\tanh ^2(y^{u^+}_{M3}-y_0)], \\ \frac{\partial \lambda ^{u^-}}{\partial u^\pm }= &  \pm \lambda _2 \, q_l \, [1-\tanh ^2(y^{u^-}_{M3}-y_0)], \\ \frac{\partial \lambda ^{u^-}}{\partial v^\pm }= &  \pm \lambda _2 \, q_l \, \alpha ^{\pm } [1-\tanh ^2(y^{u^-}_{M3}-y_0)], \\ \frac{\partial \lambda ^{v^+}}{\partial u^\pm }= &  \mp \lambda _2 \, q_l \, [1-\tanh ^2(y^{v^+}_{M3}-y_0)], \\ \frac{\partial \lambda ^{v^+}}{\partial v^\pm }= &  \mp \lambda _2 \, q_l \, \alpha ^{\pm } [1-\tanh ^2(y^{v^+}_{M3}-y_0)], \\ \frac{\partial \lambda ^{v^-}}{\partial u^\pm }= &  \pm \lambda _2 \, q_l \, [1-\tanh ^2(y^{v^-}_{M3}-y_0)], \\ \frac{\partial \lambda ^{v^-}}{\partial v^\pm }= &  \pm \lambda _2 \, q_l \, \alpha ^{\pm } [1-\tanh ^2(y^{v^-}_{M3}-y_0)], \\ \end{aligned}$$

where the perceived signal functions are given as:

$$\begin{aligned} y^{u^{\pm }}_{M3}= &  Q_l^{u^\pm }= \pm 2 \, q_l ( u^- + \alpha ^- v^- - u^+ -\alpha ^+ v^+), \\ y^{v^{\pm }}_{M3}= &  Q_l^{v^\pm }= \pm 2 \, q_l ( u^- + \alpha ^- v^- - u^+ -\alpha ^+ v^+-\frac{1}{2} \eta ). \end{aligned}$$

If at least one of the eigenvalues has a positive real part, solutions near the steady state $$S=(u^*,u^{**},v^*,v^{**})$$ diverge and the steady state is unstable. Otherwise, solutions near *S* converge to *S* and the steady state is asymptotically stable.

## Appendix B: Linear Stability Analysis of the Spatial Problem

To further assess the stability properties of the system and in particular the possibility of pattern formation, we perform a standard linear stability analysis on the *Observant* and *Persuadable* leader-only submodels, i.e. where we set $u^+=u^-=0$ in the corresponding systems of equations (Murray 1993). We note that extending the kernel $K_a$ to an odd function on the whole real line, Eqs. 17 can be written as

B3 $$\begin{aligned} Q_a^{u^\pm }= &  -q_a \int _{-\infty }^{\infty } K_a(s) u(x \pm s) ds \end{aligned}$$

B4 $$\begin{aligned} Q_a^{v^\pm }= &  -q_a \int _{-\infty }^{\infty } K_a(s) v(x \pm s) ds. \end{aligned}$$

We then set $$v^+(x,t)=v^*+ v_p(x,t)$$ and $$v^-(x,t)=v^{**}+ v_m(x,t)$$, where $$v_p(x,t)$$ and $$v_m(x,t)$$ each denote small perturbations. We substitute in the corresponding models, neglect nonlinear terms in $$v_p$$ and $$v_m$$ and look for solutions $$v_{p,m} \propto e^{\sigma t + ikx}$$. Some algebra leads to the dispersion relation of the form

B5 $$\begin{aligned} \sigma ^+(k)= \frac{C(k)+\sqrt{C^2(k)-D(k)}}{2}, \end{aligned}$$

where the expression for *C*(*k*) and *D*(*k*) slightly change for each of the models considered.

Specifically, for the *Observant* leader-only submodel (M2), we find

$$\begin{aligned} C(k)= &  (\beta _- - \beta _+)ik -2\lambda _1-\lambda _2-0.5\lambda _2 [\tanh (q_l \eta -y_0) +\tanh (-q_l\eta -y_0)], \\ D(k)= &  4 \beta _+ \beta _- k^2 +4ik\lambda _1(\beta _+-\beta _-)+ 2ik \lambda _2 \{ \beta _+(1+\tanh (q_l\eta -y_0)) \\  &  -\beta _-(1+\tanh (-q_l\eta -y_0)) +v^*(1-\tanh ^2(-q_l \, \eta -y_0))[\beta _{+} q_a {\hat{K}}_a^+ + \beta _{-} q_a {\hat{K}}_a^+] \\  &  + v^{**}(1-\tanh ^2(q_l \, \eta -y_0))[- \beta _+ q_a {\hat{K}}_a^- - \beta _- q_a {\hat{K}}_a^-] \}. \end{aligned}$$

Whereas, for the *Influenced* leader-only submodel (M3),

$$\begin{aligned} C(k)= &  (\beta _- -\beta _+)ik -2\lambda _1-\lambda _2-0.5\lambda _2 [\tanh (-2 q_l(\alpha ^- v^{**}-\alpha ^+ v^*-0.5 \eta )-y_0) \\  &  +\tanh (2 q_l(\alpha ^- v^{**}- \alpha ^+ v^*-0.5 \eta )-y_0))] \\  &  +q_l\lambda _2 ({\hat{K}}_l^+(k) +{\hat{K}}_l^-(k)) \{ v^*[1-\tanh ^2(2 q_l( \alpha ^- v^{**}-\alpha ^+ v^*-0.5 \eta )-y_0)] \\  &  + v^{**}[1-\tanh ^2(-2 q_l(\alpha ^- v^{**}-\alpha ^+ v^*-0.5 \eta )-y_0)]\}, \\ D(k)= &  4 \beta _+ \beta _- k^2 +4ik\lambda _1(\beta _+-\beta _-)+ 2ik \lambda _2 \{ \beta _+(1+\tanh (-2q_l(\alpha ^- v^{**}-\alpha ^+ v^*-0.5\eta ) -y_0)) \\  &  -\beta _-(1+\tanh (2q_l(\alpha ^- v^{**}-\alpha ^+ v^*-0.5\eta ) -y_0)) \\  &  +v^*(1-\tanh ^2(2q_l(\alpha ^- v^{**}-\alpha ^+ v^*-0.5\eta )-y_0))[\beta _+(q_a {\hat{K}}_a^+ + q_l({\hat{K}}_l^+ + {\hat{K}}_l^-)) \\  &  + \beta _-(q_a {\hat{K}}_a^+ - q_l({\hat{K}}_l^+ + {\hat{K}}_l^-))] \\  &  + v^{**}(1-\tanh ^2(-2q_l(\alpha ^- v^{**}-\alpha ^+ v^*-0.5\eta ) -y_0))[\beta _+(-q_a {\hat{K}}_a^- - q_l({\hat{K}}_l^+ + {\hat{K}}_l^-)) \\  &  + \beta _-(-q_a {\hat{K}}_a^- + q_l({\hat{K}}_l^+ + {\hat{K}}_l^-))] \}. \end{aligned}$$

In the above, ${\hat{K}}^\pm _j(k)$, for $j=a, l$ denote the Fourier transform of the kernel $K_j(s)$ defined in Eq. (7), i.e.

$$\begin{aligned} {\hat{K}}^\pm _j(k)=\int _{-\infty }^{+\infty } K_j(s) e^{\pm iks} d s = \exp \left( \pm is_j k -\frac{k^2 m_l^2}{2} \right) , \quad j=a,l. \end{aligned}$$

Figure 7 outlines the key insights that we can get from linear stability analysis of the leader-only submodels. A certain amount of attraction is crucial to aggregate a dispersed population (see Fig. 7A). Assuming sufficient attraction, the introduction of Obias generates consensus of the migration towards the target for both M2 and M3 submodels (see Fig. 7B). The M2 leader-submodel does not depend on the Cbias parameters (i.e. $\alpha ^+/\alpha ^-$). Consistently, Cbias strategy has no effect in the M2 leader-only submodel. Indeed, in the *observant* leader model Cbias only affect the alignment of the followers, which are not taken into account here. M3 leader-submodel instead generates a shift of the group orientation towards the target (see Fig. 7C). Finally, variation in the ratio $\beta _+/\beta _-$ does not alter the steady state but does affect its stability (see Fig. 7D). Excessively high values of the bias strength $\eta$, $\alpha ^+/\alpha ^-$, $\beta _+/\beta _-$ result in the population dispersing towards the uniform solution.

## Appendix C: Fixed Parameters

Table 3 summarizes the parameters set at a fixed reference value, in accordance to the previous studies in Eftimie et al. (2007) and Bernardi et al. (2021).

**Table 3:** Table of parameters kept fixed within this study

Grouping	Parameter	Description	Value [Unit]
Speed	$\gamma$	Follower speed	0.1 [L/T]
Interaction kernels	$s_l$	Alignment range	0.5 [L]
	$s_a$	Attraction range	1[L]
	$m_l$	Width of alignment kernel	0.5/8 [L]
	$m_a$	Width of attraction kernel	1/8 [L]
Turning rates	$y_0$	Shift of the turning function	2

## Appendix D: Extra Figures

See Fig. 8.
